# C3 dysregulation due to factor H deficiency is mannan-binding lectin-associated serine proteases (MASP)-1 and MASP-3 independent *in vivo*

**DOI:** 10.1111/cei.12244

**Published:** 2014-03-06

**Authors:** M M Ruseva, M Takahashi, T Fujita, M C Pickering

**Affiliations:** * Centre for Complement & Inflammation Research, Imperial College LondonLondon, UK; † Department of Immunology, Fukushima Medical University School of MedicineFukushima, Japan

**Keywords:** complement, kidney, MASP-1/3

## Abstract

Uncontrolled activation of the complement alternative pathway is associated with complement-mediated renal disease. Factor B and factor D are essential components of this pathway, while factor H (FH) is its major regulator. In complete FH deficiency, uncontrolled C3 activation through the alternative pathway results in plasma C3 depletion and complement-mediated renal disease. These are dependent on factor B. Mannan-binding lectin-associated serine proteases 1 and 3 (MASP-1, MASP-3) have been shown recently to contribute to alternative pathway activation by cleaving pro-factor D to its active form, factor D. We studied the contribution of MASP-1 and MASP-3 to uncontrolled alternative pathway activation in experimental complete FH deficiency. Co-deficiency of FH and MASP-1/MASP-3 did not ameliorate either the plasma C3 activation or glomerular C3 accumulation in FH-deficient mice. Our data indicate that MASP-1 and MASP-3 are not essential for alternative pathway activation in complete FH deficiency.

## Introduction

Complement is a crucial part of immune defence and contributes to both the innate and adaptive immune responses. It consists of a proteolytic cascade that can be triggered via the classical, lectin and alternative pathways. Complement serine proteases (reviewed in [1]) play important roles in these pathways, and include mannose-associated serine proteases (MASP-1, MASP-2, MASP-3), C1s, C1r, C2, factor B (FB) and factor D (FD). The classical pathway is activated following the interaction between C1q and immunoglobulin, while the lectin pathway is activated by the interaction of mannan-binding lectin (MBL) and ficolins with mannose or acetyl residues. These interactions trigger the activation of proteases (C1r and C1s for the classical pathway and MASPs for the lectin pathway) which, in turn, cleave C4 and C2, enabling the assembly of an enzyme complex (C4b2a) capable of cleaving C3 (C3 convertase). The alternative pathway (AP) is initiated by the spontaneous hydrolysis of C3 which, through interactions with FB and FD, generates a C3 convertase [C3(H_2_O)Bb]. These C3 convertases cleave intact C3 to generate C3b, a potent opsonin. C3b, through interactions with FB and FD, can generate more C3 convertase (C3bBb) through a positive amplification loop. Factors B and D are key components of both the AP and the C3b amplification loop. C3b binds FB forming a pro-convertase (C3bB). Within this pro-convertase, FB is cleaved by FD to generate the active convertase: C3bBb. FD is a chymotrypsin-like serine protease and FB within the pro-convertase (C3bB) is its only known natural substrate able to displace the self-inhibitory loop in the FD molecule [2,3]. The C3b amplification loop is a powerful system that can rapidly generate millions of C3b molecules. It is tightly regulated by complement factor H (FH) and complement factor I (FI). FI cleaves C3b using FH as a co-factor. Abnormalities in the function of either of these regulatory proteins results in uncontrolled C3 activation.

FH deficiency is associated with the complement-mediated kidney pathology, C3 glomerulopathy [4]. Experimental FH deficiency in pigs [5] and mice [6] results in uncontrolled AP activation characterized by low plasma C3 levels and renal disease due to abnormal accumulation of C3 within the glomeruli [6]. As would be predicted from human studies, AP activation in mice requires both FB [7] and FD [8]. Studies in FH-deficient mice (*Cfh^–/–^*, [6]) have shown that the uncontrolled AP activation requires FB: mice deficient in both FH and FB do not develop either low plasma C3 or glomerular C3 deposits [6]. This observation suggested that inhibition of AP activation could have therapeutic utility in the management of C3 glomerulopathy. This could be achieved by inhibiting either FB or FD. Although FD circulates in the blood as an active enzyme [2,9], it is synthesized in an inactive form, termed pro-factor D (pro-FD), which is devoid of proteolytic activity [10]. Cleavage of pro-FD to FD was thought to be an intracellular event mediated by endopeptidases [10–12], but murine studies indicated that this conversion required the serum proteases MASP-1 and MASP-3 [13,14]. These experiments utilized *Masp1* gene-targeted mice that, through targeted deletion of exon 2, lack both enzymatic products of the gene, MASP-1 and MASP-3, and hence are referred to as *MASP-1/3^–/–^* mice [15,16]. These animals would also be predicted to lack any murine equivalent of the truncated non-enzymatic gene product, MAp44, described in humans. These mice have impaired lectin pathway activation due to the absence of activation of MASP-2 by MASP-1 [15] and, unexpectedly, impaired activation of the AP due to the absence of conversion of pro-FD to FD by MASP-1 [13]. This latter observation raised the novel possibility that AP activation could be regulated by inhibiting MASP-1. Accordingly, in an AP-dependent arthritis model, *MASP-1/3^–/–^* mice were protected from joint inflammation, and sera from these mice showed no activity in an AP-dependent assay *in vitro* [17]. In contrast, AP activation was observed in human sera genetically deficient in both MASP-1 and MASP-3 [16]. Based on the published murine data, we hypothesized that uncontrolled AP activation in FH deficiency is dependent upon MASP-1 and/or MASP-3. To test this we intercrossed *Cfh^–/–^* and *MASP-1/3^–/–^* strains to generate mice with combined deficiency of FH, MASP-1 and MASP-3 (*Cfh^–/–^*.*MASP-1/3^–/–^*). Our *in-vivo* data demonstrated that the absence of MASP-1 and MASP-3 did not prevent uncontrolled AP activation in *Cfh^–/–^* mice. Although we observed only pro-FD in sera of *Cfh^–/–^*.*MASP-1/3^–/–^*, we were able to demonstrate FB cleavage and AP activity using murine *MASP-1/3^–/–^* sera. We conclude that MASP-1 and MASP-3 are not essential for AP activity in mice.

## Materials and methods

### Mice

The generation of *Cfh^–/–^* [6] mice and *MASP-1/3^–/–^* (*MASP1*^tm1Tefu^) [15] mice has been described previously. *Cfh^–/–^*.*MASP-1/3*^–/–^ mice were developed by intercrossing the *Cfh^–/–^* and *MASP-1/3^–/–^* strains. Breeding was monitored. Increased mortality was observed among the female breeders, leading to generation of small number of experimental animals. All mice used were on a C57BL/6 genetic background. All studies and protocols were performed in accordance with institutional guidelines and were approved by the United Kingdom Home Office.

### C3 quantification by enzyme-linked immunosorbent assay (ELISA)

Coating antibody was a goat anti-mouse C3 polyclonal antibody (product no. 55463; MP Biomedicals LLC, Strasbourg, France) used at a dilution of 1:8000 in 0·1 M carbonate buffer, pH 9·6. Captured mouse C3 was detected using a horseradish peroxidase (HRP)-conjugated goat anti-mouse C3 polyclonal antibody (product no. 55557; MP Biomedicals) used at a dilution of 1:25 000 phosphate-buffered saline (PBS)/0·2% Tween. Plates were developed using 3,3′,5,5′-tetramethylbenzidine (TMB) substrate (Sigma-Aldrich, Poole, UK). Concentration of plasma C3 was estimated by reference to a calibration curve constructed using reference sera containing a known amount of mouse C3 (serum amyloid P mouse standard, product no. 565193; Calbiochem, Darmstadt, Germany).

### Western blot analysis of mouse C3, C5 and FB

Mouse blood was collected by cardiac puncture in the presence of ethylenediamine tetraacetic acid (EDTA), chilled promptly on ice and the plasma separated. The proteins were separated using sodium dodecyl sulphate-polyacrylamide gel electrophoresis (SDS-PAGE): 7·5% gel under non-reducing conditions for C5 and 10% gel under reducing conditions for C3 and FB analysis. The Western blot membranes were blocked in 5% w/v non-fat dry milk/PBS. The same buffer was used for diluting the detection and secondary antibodies. Detection antibodies were: goat anti-serum to mouse C3 (product no. 55444; MP Biomedicals), goat anti-serum to human factor B (product no. A311; Quidel, San Diego, USA) and goat anti-serum to human C5 (product no. A306; Quidel). Secondary antibody was HRP-conjugated anti-goat immunoglobulin (product no. A9452; Sigma-Aldrich). Blots were visualized using Pierce enhanced chemiluminescence (ECL) Western blotting substrate (Thermo Scientific, Erembogdegem, Aalst, Belgium).

### Immunoprecipitation and Western blot analysis of FD

Mouse plasma (25 μl) was incubated for 1 h with either a polyclonal affinity-purified goat anti-mouse FD antibody (2·8 μg, product no. AF5430; R&D Systems, Abingdon, UK) or an immunoglobulin (IgG) fraction of polyclonal goat anti-mouse C3 (2·8 μg, product no. 55463; MP Biomedicals). Samples then were mixed with 12 μl protein A/G PLUS agarose (Santa Cruz Biotechnology, Santa Cruz, CA, USA). The reaction was incubated overnight at 4°C. Beads were washed with PBS and sample denatured at 100°C for 5 min with glycoprotein denaturing buffer (New England Biolabs, Hitchin, UK). Denatured reaction was incubated with peptide-N-glycosidase F (product no. P0704S; New England Biolabs) for 1 h at 37°C. The samples were centrifuged and the supernatant separated. FD was detected using Western blot with a biotinylated anti-mouse FD polyclonal antibody (product no. BAF5430; R&D Systems) and streptavidin–HRP (product no. P0397; Dako, Glostrup, Denmark). The proteins were visualized using Pierce ECL Western blotting substrate (Thermo Scientific).

### Immunostaining

Cryosections (5 μm) from snap-frozen kidneys were fixed in acetone for 10 min. C3 was visualized using a fluorescein isothiocyanate (FITC)-conjugated goat anti-mouse C3 polyclonal antibody (product no. 55500; MP Biomedicals). Podocytes were visualized using a biotinylated hamster anti-mouse podoplanin monoclonal antibody (product no. 13-5381-82; eBioscience, Hatfield, UK) and a streptavidin Alexa Fluor 555 (Life Technologies, Paisley, UK). Quantitative immunofluorescence staining was performed as described previously [18]. Ten glomeruli were assessed per section and fluorescence intensity expressed in arbitrary units. Tubulo-interstitial C3 deposition was scored in a blinded manner using a categorical scoring scale defined as: absent staining = 0, reduced staining = 1, comparable staining to wild-type mice = 2.

### Measurement of serum AP activity

Mouse blood was collected, allowed to clot on ice, serum separated and assayed immediately. The serum was diluted in AP buffer [5 mM sodium barbitone, pH 7·4, 150 mM NaCl, 10 mM ethylene glycol-bis-(β-aminoethyl ether) *N*,*N*,*N*′,*N*′-tetraacetic acid (EGTA), 7 mM MgCl_2_, 0·1% (w/v) gelatin] and 1 : 2 dilution series were set up. Rabbit erythrocytes were washed and resuspended to 1% (v/v) in to AP buffer. Twenty-five μl of erythrocytes suspension was incubated with 50 μl of each serum concentration for 1 h at 37°C. The absorbance of the supernatant was measured at 415 nm and percentage haemolysis was calculated by standard methods [19].

### Administration of cobra venom factor (CVF)

MASP-1/3-deficient (*n* = 3) and wild-type (*n* = 3) male mice were injected with 30 μg CVF (product no. A600; Quidel) intraperitonally. EDTA–plasma samples were collected prior to and 2 and 8 h post-administration. FB activation was analysed by Western blot. Plasma C3 was assessed by Western blot and ELISA.

## Results

### Deficiency of MASP-1 and MASP-3 does not alter C3 and C5 levels in factor H-deficient mice

**Figure 1:**
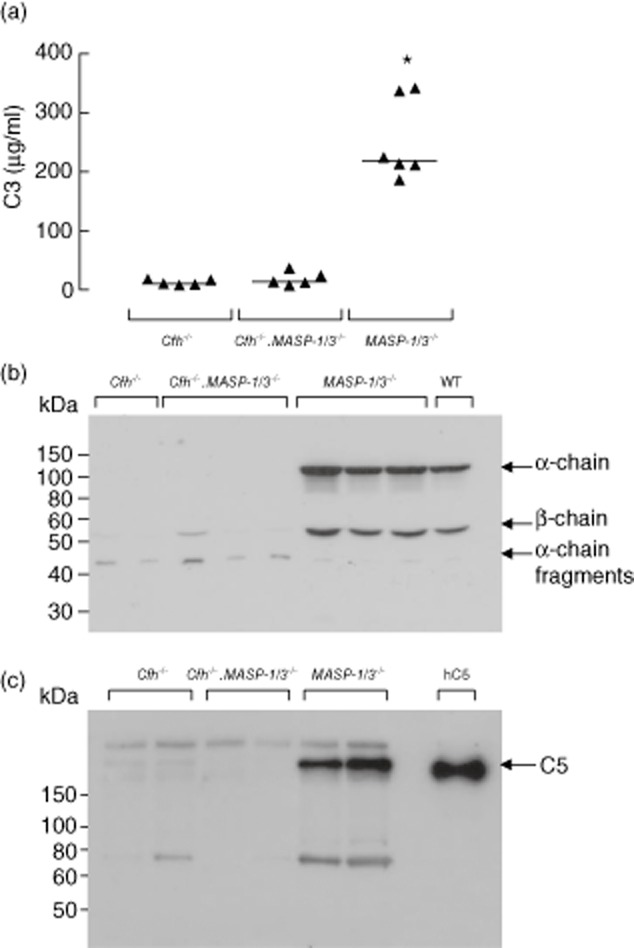
Plasma C3 and C5 in *Cfh^–/–^*, *Cfh^–/–^*.mannan-binding lectin-associated serine proteases (*MASP*)*-1/3^–/–^* and *MASP-1/3^–/–^* mice. (a) C3 levels were measured by enzyme-linked immunosorbent assay (ELISA). C3 levels were within the normal range in *MASP-1/3^–/–^* mice, while these were reduced to a comparable extent in both *Cfh^–/–^* and *Cfh^–/–^*.*MASP-1/3^–/–^* animals. Horizontal bars denote median values. *P < 0·001 *versus* either *Cfh^–/–^* or *Cfh^–/–^*.*MASP-1/3^–/–^* groups, Bonferroni's multiple comparison test. (b) Western blot for C3 under reducing conditions using plasma from *Cfh^–/–^*, *Cfh^–/–^*.*MASP-1/3^–/–^*, *MASP-1/3^–/–^* and wild-type mice. Intact C3 α-chain and β-chain were seen in *MASP-1/3^–/–^* and wild-type mice. In contrast, intact α-chain was absent in *Cfh^–/–^* and *Cfh^–/–^*.*MASP-1/3^–/–^*. In both strains, α-chain fragments were detected. (c) C5 was analysed by Western blot under non-reducing conditions using 1 μl of mouse plasma. Under these conditions C5 was readily detectable in *MASP-1/3^–/–^* mice, while it was barely visible or absent in *Cfh^–/–^* and *Cfh^–/–^*.*MASP-1/3^–/–^* animals. Purified human C5 (hC5) was used as control. Non-specific bands were detected in the mouse plasma samples but not in the human C5 preparation.

Plasma C3 and C5 levels are severely reduced in *Cfh^–/–^* mice due to uncontrolled AP activation [6,20,21]. To assess the contribution of MASP-1 and MASP-3 to this phenotype we measured plasma C3 levels in *Cfh^–/–^*.*MASP-1/3^–/–^* animals (Fig. 1a). Plasma C3 was markedly decreased to a comparable degree in both *Cfh^–/–^*.*MASP-1/3^–/–^* (median = 13·3 μg/ml, range 7·1–36, *n* = 5) and *Cfh^–/–^* (median = 10 μg/ml, range 7–18, *n* = 5) animals. In contrast, C3 levels were normal in *MASP-1/3^–/–^* mice (median = 218·6 μg/ml, range 185·8–341·8, *n* = 6, *P* < 0·05 *versus* either *Cfh^–/–^*.*MASP-1/3^–/–^* or *Cfh^–/–^* groups, Bonferroni's multiple comparison test). Analysis of plasma C3 by Western blotting under reduced conditions showed absence of intact C3 α-chain in both *Cfh^–/–^*.*MASP-1/3^–/–^* and *Cfh^–/–^* mice (Fig. 1b). We assessed circulating C5 levels in *Cfh*^–/–^.*MASP-1/3*^–/–^ mice by Western blotting of plasma under non-reducing conditions (Fig. 1c). Plasma C5 was readily detectable in *MASP-1/3^–/–^* plasma, but absent or barely visible in *Cfh^–/–^*.*MASP-1/3^–/–^* and *Cfh^–/–^* plasma samples. Therefore, both the plasma C3 and C5 depletion associated with FH deficiency is unaffected by the absence of MASP-1 and MASP-3.

### Deficiency of MASP-1 and MASP-3 does not ameliorate glomerular C3 accumulation in factor H-deficient mice

**Figure 2:**
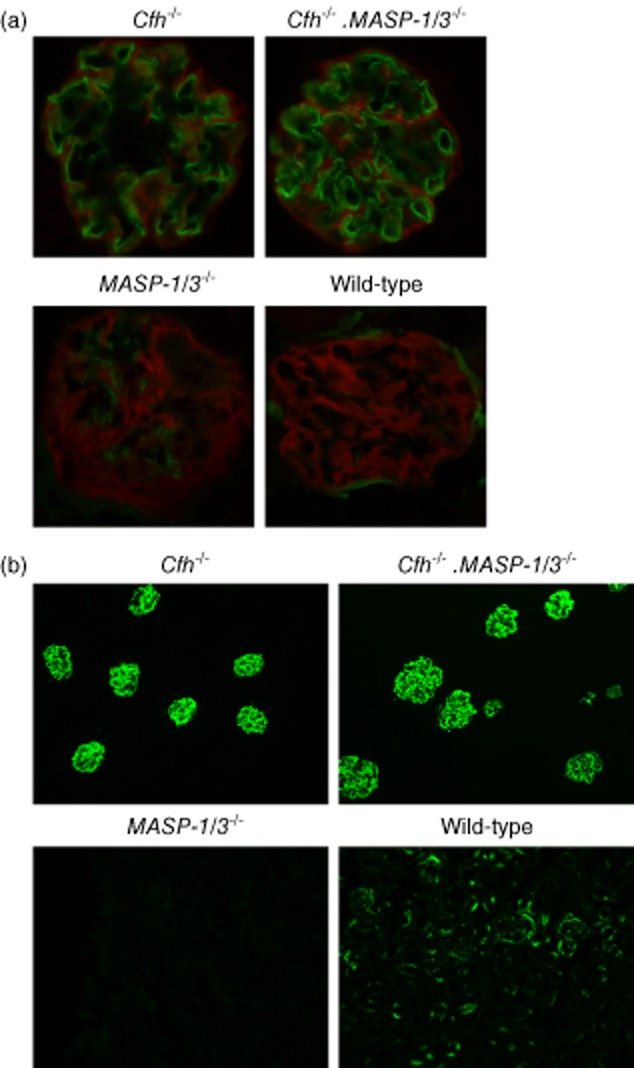
C3 reactivity in kidneys from *Cfh^–/–^*, *Cfh^–/–^*.mannan-binding lectin-associated serine proteases (*MASP)-1/3^–/–^*, *MASP-1/3^–/–^* and wild-type mice. (a) Representative images of glomerular C3 reactivity. Kidney sections were stained with anti-C3 antibody (green) and anti-podoplanin antibody (to detect podocytes, red). Similar to the *Cfh^–/–^* mice, *Cfh^–/–^*.*MASP-1/3^–/–^* sections had linear C3 staining beneath the podocyte layer consistent with C3 deposition along the glomerular basement membrane (GBM). Little or no C3 was detected in the glomeruli from *MASP-1/3^–/–^* animals. As expected, no glomerular C3 reactivity was evident in the wild-type mice. The average diameter of mouse glomerulus was approximately 60 microns. Original magnification ×40. (b) Representative images of tubulo-interstitial C3 reactivity. As demonstrated previously, no tubulo-interstitial C3 staining was evident in *Cfh^–/–^* mice. No staining was evident in both *Cfh^–/–^*.*MASP-1/3^–/–^* and *MASP-1/3^–/–^* mice. Original magnification ×20.

*Cfh^–/–^* mice have abnormal accumulation of glomerular C3 along the glomerular basement membrane (GBM) [6]. *Cfh^–/–^*.*MASP-1/3^–/–^* animals developed identical GBM C3 accumulation to that seen in *Cfh^–/–^* animals (Fig. 2a). No difference in intensity of the staining was observed between these two groups: median fluorescence intensity was 1519 (range of values: 953·1–2088, *n* = 5) and 1763 (range of values: 1641–2074, *n* = 4) in *Cfh^–/–^* and *Cfh^–/–^*.*MASP-1/3^–/–^*, respectively, *P* > 0·05, Mann–Whitney *U*-test. A minor level of glomerular C3 reactivity with a granular pattern of distribution was evident in two of the *MASP-1/3^–/–^* animals, while in the other three animals examined glomerular C3 was absent. Tubulo-interstitial C3 staining, an AP-dependent phenomenon [22–24], is seen normally in wild-type mice, but absent in *Cfh^–/–^* animals (Fig. 2b). Tubulo-interstitial C3 staining was absent in all *Cfh^–/–^*.*MASP-1/3^–/–^* animals examined (*n* = 5, median tubulo-interstitial staining score = 0). Using this scale, tubulo-interstitial staining scores in wild-type mice (*n* = 5) were all graded 2 (see Methods).

### Pro-FD is detectable in plasma from *Cfh^–/–^*.*MASP-1/3^–/–^* mice

**Figure 3:**
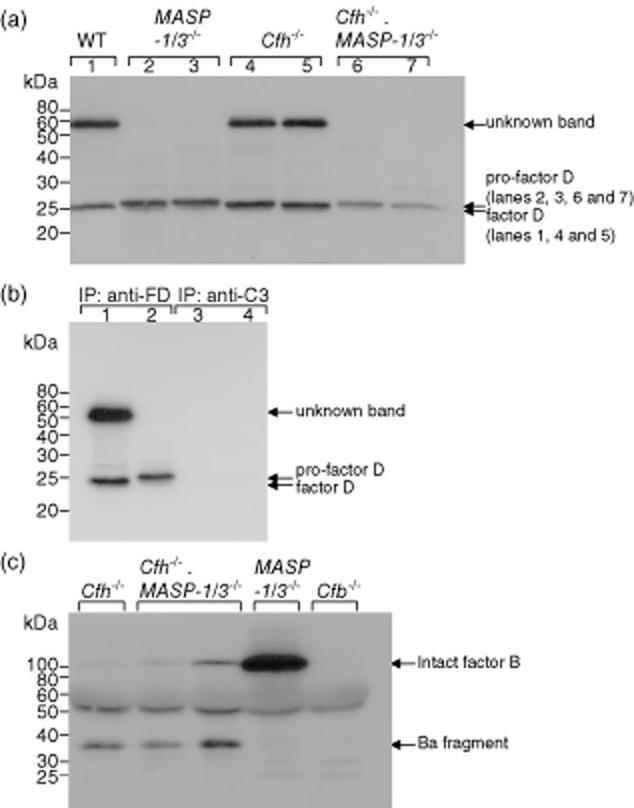
Western blot analysis of factor D (FD) and factor B (FB) in *Cfh^–/–^*, *Cfh^–/–^*.mannan-binding lectin-associated serine proteases (*MASP)-1/3^–/–^* and *MASP-1/3^–/–^* mice. (a) FD in mouse plasma was immunoprecipitated with goat anti-FD polyclonal antibody, incubated with N-glycosidase and analysed by immunoblotting. Evidence of pro-FD was seen in *MASP-1/3^–/–^* (lanes 2 and 3) and *Cfh^–/–^*.*MASP-1/3^–/–^* (lanes 6 and 7) mice. In wild-type and *Cfh^–/–^* animals FD was evident in addition to an unidentified higher molecular weight band. The Western blot was not designed to allow FD quantification. (b) Plasma from wild-type (lanes 1 and 3) and *MASP-1/3^–/–^* mice (lanes 2 and 4) was subjected to immunoprecipitation with goat anti-FD (lanes 1 and 2) or goat anti-C3 (lanes 3 and 4) polyclonal antibodies. The immunoprecipitated proteins were incubated with N-glycosidase and analysed for FD by immunoblotting. FD-related bands were observed after immunoprecipitation with goat anti-FD antibody (lanes 1 and 2) and no cross-reactivity with mouse or goat immunoglobulins was evident (lanes 3 and 4). IP: immunoprecipitation. (c) FB was analysed by Western blotting under reduced conditions. Intact FB and Ba fragments were detected using anti-human FB polyclonal serum that cross-reacts with mouse FB. The Bb fragment could not be assessed accurately, as a background band with similar molecular weight (∼55 kDa) was observed in the *Cfb^–/–^*. Data are representative of two independent experiments.

It has been shown previously that mouse MASP-1 and MASP-3 cleave the N-terminal five amino acid activation peptide (QPRGR) of pro-FD to generate the FD [13,14]. Consistent with this, pro-FD is present in plasma from *MASP-1/3^–/–^* animals [13,14,17]. To determine the state of FD in *Cfh^–/–^*.*MASP-1/3^–/–^* animals we performed immunoprecipitation with plasma samples using a goat anti-mouse FD antibody that recognizes both pro-FD and FD. Immunoprecipitated FD was then treated with N-glycosidase F to remove N-glycosylation modifications [13]. This de-glycosylation has been shown to alter the Western blot appearance of FD from a broad molecular weight range of 40–44 kDa to a single 26 kDa band, thereby allowing pro-FD and factor FD to be distinguished by size [13]. Enzymatically de-glycosylated FD from *Cfh^–/–^* and wild-type samples was detectable (Fig. 3a) and had the expected molecular weight of ∼26 kDa [13]. In both *MASP-1/3^–/–^* and *Cfh^–/–^*.*MASP-1/3^–/–^* samples FD was also detectable, but the predominant band ran slightly higher than the 25 kDa band seen in the *Cfh^–/–^* and wild-type samples (Fig. 3a). We interpreted this higher band to be pro-FD based on previous data using this procedure in the *MASP-1/3^–/–^* animals [13]. A further difference between the samples from the wild-type and *Cfh^–/–^* group *versus* the *MASP1/3^–/–^* and *Cfh^–/–^*.*MASP1/3^–/–^* group was the finding of a higher molecular weight band in the *Cfh^–/–^* and wild-type samples (lanes 1, 4 and 5, Fig. 3a). Previous data have also shown this finding, and it has been interpreted as a complex between mature FD and, to date, an unidentified serum protein [12,17]. Whether or not this interpretation is correct, this high molecular weight band was absent in sera from both *MASP-1/3^–/–^* and *Cfh^–/–^*.*MASP-1/3^–/–^* animals, indicating that this phenomenon does not occur for pro-FD. Importantly, this higher molecular weight band was absent when a polyclonal goat anti-mouse C3 antibody was used in place of the polyclonal goat anti-mouse FD during immunoprecipitation (Fig. 3b). This excluded the possibility that this band represented cross-reactivity of the anti-FD developing antibody with goat and mouse immunoglobulins in the reaction (Fig. 3b).

### Activated factor B is detectable in plasma in the absence of MASP-1 and MASP-3

**Figure 4:**
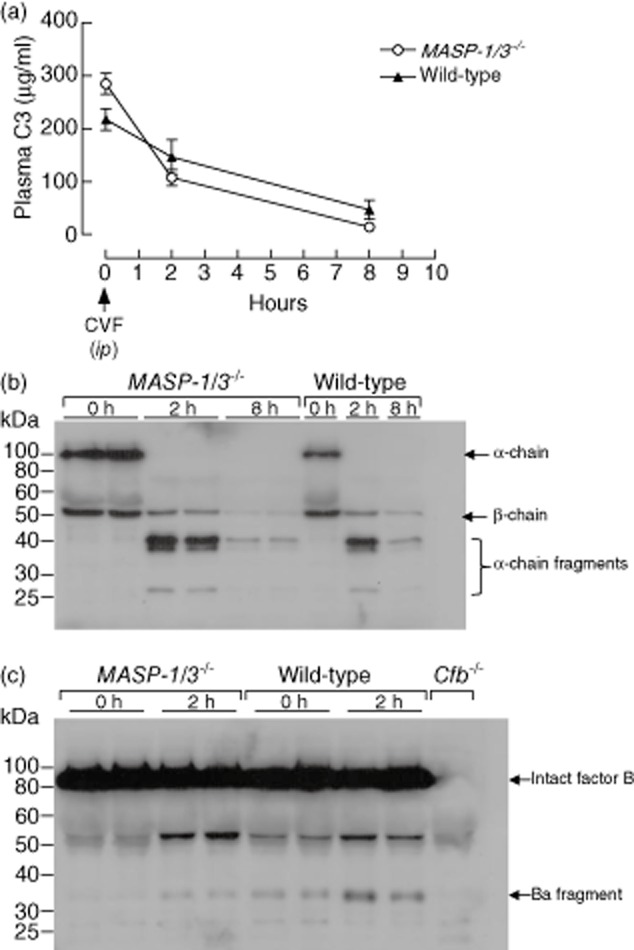
Activation of C3 and factor B (FB) by cobra venom factor (CVF). Mannan-binding lectin-associated serine protease (*MASP)-1/3^–/–^* and wild-type mice were given a single intraperitoneal 30 μg dose of CVF. (a) Plasma C3 levels prior (0 h) and post (2 h and 8 h)-treatment were measured by enzyme-linked immunosorbent assay (ELISA) and data points represent mean ± standard deviation (s.d.) of three mice in each group. (b) CVF-mediated C3 activation was assessed by Western blot analysis under reducing conditions. No intact C3 α-chain was detected after treatment in plasma from *MASP-1/3^–/–^* (two mice shown) and wild-type animals (one mouse shown). C3 activation products were evident in both groups. (c) FB activation was assessed by Western blot analysis under reducing conditions. Ba fragment was evident in plasma of *MASP-1/3^–/–^* and wild-type mice 2 h after administration of CVF to a greater extent than prior to injection. As in Fig. 3c, the background reactivity with the anti-factor B antibody was demonstrated using FB-deficient plasma obtained from gene-targeted FB-deficient mice (*Cfb^–/–^*).

The physiological substrate of FD is FB complexed with C3b (C3bB), a structure termed the C3 pro-convertase. FD cleaves the Arg234-Lys235 bond in FB to generate C3bBb releasing a 234 amino acid FB fragment termed Ba [25–27]. It has been reported that pro-FD, in contrast to FD, is proteolytically inactive [10]. Our data demonstrated that FD in *Cfh^–/–^*.*MASP-1/3^–/–^* mice was in the form of pro-FD (Fig. 3a), yet these animals were C3-depleted (Fig. 1). We next looked at the activation state of circulating FB by Western blot analysis of plasma under non-reducing conditions. In FH deficiency, impaired control of the AP C3 convertase is associated with abnormal activation of AP proteins in the fluid phase. As expected, factor B activation was evident in *Cfh^–/–^* mice. FB activation fragment Ba was also present in *Cfh^–/–^*.*MASP1/3^–/–^* mice (Fig. 3c). The Bb fragment could not be assessed accurately, as a background band with similar molecular weight (∼55 kDa) was observed in the FB-deficient mice (*Cfb^–/–^*). To further determine if canonical cleavage of FB could proceed in MASP-1/3-deficient sera *in vivo* we administered CVF to wild-type and *MASP-1/3^–/–^* mice. The CVF-mediated cleavage of C3 has been shown to require FB, but not factor FD [8]. However, in FD-deficient mouse serum treated with CVF *in vitro* no cleavage of FB occurs. Based on these published data, we next determined if FB cleavage occurred in *MASP-1/3^–/–^* mice treated with CVF (Fig. 4). In these animals, CVF treatment resulted in C3 depletion with concomitant cleavage of FB similar to that seen in CVF-treated wild-type mice.

Assuming that pro-FD is devoid of proteolytic activity, these findings indicated that there was (i) active FD in unmanipulated *Cfh^–/–^*.*MASP-1/3^–/–^* mice, (ii) active FD in CVF-treated *MASP-1/3^–/–^* mice and (iii) MASP-1/3-independent conversion of pro-FD to FD in both groups. However, we could not definitively visualize mature FD using Western blotting techniques with MASP-1/3-deficient sera (lanes 2, 3, 6 and 7, Fig. 3a).

### Alternative pathway haemolytic activity is reduced but not absent in *MASP-1/3^–/–^* mice

**Figure 5:**
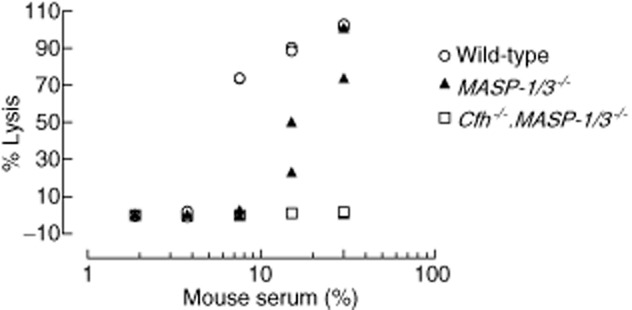
Serum alternative pathway activity in *Cfh^–/–^*.mannan-binding lectin-associated serine proteases (*MASP)-1/3^–/–^* and *MASP-1/3^–/–^* mice. Rabbit erythrocytes were incubated with dilutions of serum from *Cfh^–/–^*.*MASP-1/3^–/–^* and *MASP-1/3^–/–^* and wild-type mice (*n* = 2 per group) and haemolytic activity was assessed by measuring release of haemoglobin in to the supernatant. Percentage lysis was plotted against serum concentration (expressed as percentage, logarithmic scale). Each symbol represents percentage lysis from an individual mouse and each point is an average of duplicate measurements. Data from one of two independent experiments with similar results are shown.

Using a calcium-free rabbit erythrocyte haemolysis *in-vitro* assay, AP-mediated cell lysis was readily detectable with wild-type sera (Fig. 5). As expected, no lysis was demonstrable using sera from *Cfh^–/–^*.*MASP-1/3^–/–^* mice, as these sera are depleted of both C3 and C5 (Fig. 1). However, sera from *MASP-1/3^–/–^* mice, in contrast to previous reported *in-vitro* assays [13,17], did demonstrate lysis, although this was reduced in comparison with that of wild-type mice.

## Discussion

Our investigation was initiated following the report that *MASP-1/3^–/–^* mice had absent AP activation due to an essential role for MASP-1 in the conversion of pro-FD to FD [13]. Using either a rabbit haemolytic or zymosan plate assay, no AP activity was demonstrable in sera from *MASP-1/3^–/–^* mice [13]. We hypothesized that uncontrolled AP activation due to FH deficiency might be prevented by the absence of MASP-1 because of an inability to convert pro-FD to FD. Notably, we have shown previously that the absence of FB prevented uncontrolled C3 activation *Cfh^–/–^* mice [6]. Our present data demonstrated that co-deficiency of MASP-1 and MASP-3 did not prevent uncontrolled AP activation in the setting of complete FH deficiency.

The *Cfh^–/–^*.*MASP-1/3^–/–^* animals had comparable plasma C3 depletion and glomerular C3 staining intensities to mice deficient in FH alone. We interpreted this to indicate that there was mature FD in the *Cfh^–/–^*.*MASP-1/3^–/–^* animals. However, when we examined immunoprecipitated and glycosidase-treated FD in these animals by Western blotting the appearances, similar in both *Cfh^–/–^*.*MASP-1/3^–/–^* and *MASP-1/3^–/–^* animals, revealed the presence of pro-FD but not FD (Fig. 3a). In previous studies, only pro-FD was detected in *MASP-1/3^–/–^* animals both spontaneously [13] and during experimental inflammation [17]. However, differentiating pro-FD and FD is technically challenging *in vivo*, as the two differ only by a five amino acid activation peptide (QPRGR [13]). We therefore took a different approach and looked for *in-vivo* evidence of FB activation. The finding of cleavage fragments of FB, namely Bb and/or Ba, would provide indirect evidence for the presence of FD, as pro-FD is unable to cleave FB [13,28]. We were able to detect FB cleavage in the setting of MASP-1 and MASP-3 co-deficiency in two situations. First, we were able to see Ba fragments in plasma of *Cfh^–/–^*.*MASP-1/3^–/–^* animals to an equivalent extent to that seen in *Cfh^–/–^* mice (Fig. 3c). Secondly, Ba fragments increased in *MASP1/3^–/–^* mice after the administration of CVF (Fig. 4c). This latter approach is the *in-vivo* counterpart to an experiment performed *in vitro* in which purified components were used to detect FB cleavage [13]. Cleavage of FB was seen when recombinant mouse pro-FD was incubated with purified human factor B, CVF and recombinant mouse MASP-1 (rMASP-1K, in which arginine 429 is replaced with lysine [15]) [13]. In contrast, no cleavage was seen when recombinant mouse pro-FD was incubated with purified human FB and CVF in the absence of rMASP-1K, demonstrating that pro-FD is unable to cleave factor B bound to CVF. It is important to note that activation of C3 in mice by CVF is independent of the presence of FD: plasma C3 depletion occurred in both wild-type and FD-deficient mice following administration of CVF, although the depletion was slower in the absence of FD [8]. These data indicated that both CVF-Bb and CVF-B are able to cleave C3 in mice. Hence, our finding that plasma C3 depleted after administration of CVF to *MASP-1/3^–/–^* mice provided no information on FD status, as this would be expected whether FD was absent, present as pro-FD or present as FD. However, and key to the interpretation of our data, the CVF-induced cleavage of FB is absolutely dependent upon the presence of FD [8]. Furthermore, fluid-phase FB activation was demonstrated only when pro-FD was activated with trypsin [28]. Hence, our demonstration that FB cleavage occurred after administration of CVF to *MASP-1/3^–/–^* mice indicated that some FD must be present *in vivo*.

If there were some FD present in *MASP-1/3^–/–^* mice we would expect to be able to demonstrate some AP activity. Using a haemolytic assay we were able to detect AP-mediated lysis using sera from *MASP1/3^–/–^* mice, although this was lower than that seen in wild-type mice using identical concentrations of sera. We concluded that AP activity is impaired but not absent in these animals. It is possible that abnormalities in adipose tissue contribute to this, as fat atrophy has been reported in this strain [29] and FD is synthesized by adipocytes [30]. Additional evidence for a role for MASP-1 in AP activation derived from studies in which the *MASP-1/3^–/–^* mice have been subjected to an AP-dependent model of arthritis [17]. In this collagen antibody arthritis model the AP is both necessary and sufficient [31,32]. Although, to our knowledge, FB and *MASP-1/3^–/–^* mice have not been compared directly in this model, from the reported data sets it appears that mice with FB deficiency have almost absent histopathology scores [31], while *MASP-1/3^–/–^* mice develop reduced histopathology scores when compared to wild-type [17]. This would be consistent with impaired but not absent AP in *MASP-1/3^–/–^* mice. Alternatively, it could be that the pre-existing reduction in AP activation in *MASP-1/3^–/–^* mice was regulated sufficiently by FH *in vivo* in order to prevent full manifestation of the AP-mediated pathology in this model.

A further finding that suggested impaired AP activation in *MASP-1/3^–/–^* mice *in vivo* was the pattern of renal C3 immunostaining seen in unmanipulated *MASP-1/3^–/–^* animals. This was qualitatively similar to that of wild-type mice but markedly reduced in intensity (Fig. 2). In wild-type mice, staining for C3 along Bowman's capsule and tubular cells is typical. This tubulo-interstitial C3 staining is AP-dependent, specifically on an intact AP pathway in circulation [23,33]. Hence, tubulo-interstitial C3 is absent in *Cfh^–/–^* mice [6,24], but reappears when systemic AP activity is restored, either temporarily after the administration of mouse [34] or human [35] FH or long-term through renal transplantation [33].

What is the relevance of these murine phenotypes to human complement biology? The human *MASP1* gene encodes for MASP-1, MASP-3 and the non-enzymatic protein, MAp44. Recently, a patient with a *MASP1* gene mutation that results in the absence of all three products (MASP-1, MASP-3 and MAp44) has been described [16,36]. Using sera from this patient, lectin pathway was absent but reconstituted with recombinant MASP-1 [16]. Similarly, lectin pathway was impaired in *MASP-1/3^–/–^* mice and restored with recombinant MASP-1 [15]. However, when AP activity was compared between human and mice with co-deficiency of MASP-1 and MASP-3 apparent differences emerged [13,16]. In the single human with co-deficiency of MASP-1 and MASP-3, AP activity was considered to be intact [16]. The status of FD (pro-FD *versus* FD) was not assessed but, as we have carried out in the present paper, it could be inferred that some FD was present because AP activity was demonstrable *in vitro* [16,37]. As this was a single patient and the normal range of AP activity in humans is broad, it was not possible to determine if the AP activity in the patient was within or below the normal range. Nevertheless, it can be robustly concluded that AP activation was possible in the absence of both MASP-1 and MASP-3. Although reported originally as absent [13], under the assay conditions utilized in this report we could detect AP activity, albeit reduced, in murine sera lacking MASP-1 and MASP-3. Furthermore, MASP-1 has been shown to cleave both human [38] and mouse [13] pro-FD to FD. At present, it seems likely that there is a non-essential role for MASP-1 on the FD functional activity in mice and humans. In the absence of MASP-1 other enzymes, e.g. kallikrein and plasmin [38], mediate this cleavage, enabling the production of some FD and preserving some degree of AP activation.

A separate issue that arose during this study was the observation that the maintenance of the *MASP-1/3^–/–^* mice was problematic due to increased mortality among the breeding animals. We noticed increased mortality specifically among the female breeders resulting in frequent loss of young pups. Mutations in the human *MASP1* gene mutation have been defined as the cause of the developmental disorder: Malpeuch–Michels–Mingarelli–Carnevale (3MC) syndrome [39]. This indicated a critical role for MASP-1 and MASP-3 in development. Recently, developmental analysis of the *MASP-1/3^–/–^* mice has shown that they develop skeletal abnormalities [40]. This, together with other developmental problems, may account for the breeding difficulties we encountered and could also potentially influence the pathology when these mice are used in certain disease models.

In summary, we have demonstrated that uncontrolled AP activation in murine FH deficiency is not altered by concomitant deficiency of MASP-1 and MASP-3. The implication is that inhibition of MASP-1 is not a viable strategy to treat renal disease associated with uncontrolled AP activation. Our data also demonstrate that AP activation can occur in the absence of MASP-1, MASP-3 and FH *in vivo*.

## References

[1] Forneris F, Wu J, Gros P (2012). The modular serine proteases of the complement cascade. Curr Opin Struct Biol.

[2] Jing H, Babu YS, Moore D (1998). Structures of native and complexed complement factor D: implications of the atypical His57 conformation and self-inhibitory loop in the regulation of specific serine protease activity. J Mol Biol.

[3] Forneris F, Ricklin D, Wu J (2010). Structures of C3b in complex with factors B and D give insight into complement convertase formation. Science.

[4] Fakhouri F, Frémeaux-Bacchi V, Noël LH, Cook HT, Pickering MC (2010). C3 glomerulopathy: a new classification. Nat Rev Nephrol.

[5] Høgåsen K, Jansen JH, Mollnes TE, Hovdenes J, Harboe M (1995). Hereditary porcine membranoproliferative glomerulonephritis type II is caused by factor H deficiency. J Clin Invest.

[6] Pickering MC, Cook HT, Warren J (2002). Uncontrolled C3 activation causes membranoproliferative glomerulonephritis in mice deficient in complement factor H. Nat Genet.

[7] Matsumoto M, Fukuda W, Circolo A (1997). Abrogation of the alternative complement pathway by targeted deletion of murine factor B. Proc Natl Acad Sci USA.

[8] Xu Y, Ma M, Ippolito GC, Schroeder HW, Carroll MC, Volanakis JE (2001). Complement activation in factor D-deficient mice. Proc Natl Acad Sci USA.

[9] Jing H, Macon KJ, Moore D, DeLucas LJ, Volanakis JE, Narayana SV (1999). Structural basis of profactor D activation: from a highly flexible zymogen to a novel self-inhibited serine protease, complement factor D. EMBO J.

[10] Yamauchi Y, Stevens JW, Macon KJ, Volanakis JE (1994). Recombinant and native zymogen forms of human complement factor D. J Immunol.

[11] Lesavre PH, Müller-Eberhard HJ (1978). Mechanism of action of factor D of the alternative complement pathway. J Exp Med.

[12] Rosen BS, Cook KS, Yaglom J (1989). Adipsin and complement factor D activity: an immune-related defect in obesity. Science.

[13] Takahashi M, Ishida Y, Iwaki D (2010). Essential role of mannose-binding lectin-associated serine protease-1 in activation of the complement factor D. J Exp Med.

[14] Iwaki D, Kanno K, Takahashi M, Endo Y, Matsushita M, Fujita T (2011). The role of mannose-binding lectin-associated serine protease-3 in activation of the alternative complement pathway. J Immunol.

[15] Takahashi M, Iwaki D, Kanno K (2008). Mannose-binding lectin (MBL)-associated serine protease (MASP)-1 contributes to activation of the lectin complement pathway. J Immunol.

[16] Degn SE, Jensen L, Hansen AG (2012). Mannan-binding lectin-associated serine protease (MASP)-1 is crucial for lectin pathway activation in human serum, whereas neither MASP-1 nor MASP-3 is required for alternative pathway function. J Immunol.

[17] Banda NK, Takahashi M, Levitt B (2010). Essential role of complement mannose-binding lectin-associated serine proteases-1/3 in the murine collagen antibody-induced model of inflammatory arthritis. J Immunol.

[18] Robson MG, Cook HT, Botto M (2001). Accelerated nephrotoxic nephritis is exacerbated in C1q-deficient mice. J Immunol.

[19] Morgan BP (2000). Measurement of complement hemolytic activity, generation of complement-depleted sera, and production of hemolytic intermediates. Methods Mol Biol.

[20] Ruseva MM, Vernon KA, Lesher AM (2013). Loss of properdin exacerbates C3 glomerulopathy resulting from factor H deficiency. J Am Soc Nephrol.

[21] de Jorge EG, Macor P, Paixão-Cavalcante D (2011). The development of atypical hemolytic uremic syndrome depends on complement C5. J Am Soc Nephrol.

[22] Nath KA, Hostetter MK, Hostetter TH (1985). Pathophysiology of chronic tubulo-interstitial disease in rats. Interactions of dietary acid load, ammonia, and complement component C3. J Clin Invest.

[23] Thurman JM, Ljubanovic D, Edelstein CL, Gilkeson GS, Holers VM (2003). Lack of a functional alternative complement pathway ameliorates ischemic acute renal failure in mice. J Immunol.

[24] Quigg RJ, Lim A, Haas M, Alexander JJ, He C, Carroll MC (1998). Immune complex glomerulonephritis in C4-and C3-deficient mice. Kidney Int.

[25] Morley BJ, Campbell RD (1984). Internal homologies of the Ba fragment from human complement component Factor B, a class III MHC antigen. EMBO J.

[26] Niemann MA, Volanakis JE, Mole JE (1980). Amino-terminal sequence of human factor B of the alternative complement pathway and its cleavage fragments, Ba and Bb. Biochemistry.

[27] Taylor FR, Bixler SA, Budman JI (1999). Induced fit activation mechanism of the exceptionally specific serine protease, complement factor D. Biochemistry.

[28] Fearon DT, Austen KF, Ruddy S (1974). Properdin factor D: characterization of its active site and isolation of the precursor form. J Exp Med.

[29] Minoru T, Daisuke I, Yuichi E, Teizo F, Abdelmohsen K (2012). The Study of MASPs Knockout Mice. Binding protein.

[30] Cook KS, Groves DL, Min HY, Spiegelman BM (1985). A developmentally regulated mRNA from 3T3 adipocytes encodes a novel serine protease homologue. Proc Natl Acad Sci USA.

[31] Banda N, Thurman J, Kraus D (2006). Alternative complement pathway activation is essential for inflammation and joint destruction in the passive transfer model of collagen-induced arthritis. J Immunol.

[32] Banda N, Takahashi K, Wood A, Holers V, Arend W (2007). Pathogenic complement activation in collagen antibody-induced arthritis in mice requires amplification by the alternative pathway. J Immunol.

[33] Alexander JJ, Wang Y, Chang A (2007). Mouse podocyte complement factor H: the functional analog to human complement receptor 1. J Am Soc Nephrol.

[34] Paixão-Cavalcante D, Hanson S, Botto M, Cook H, Pickering M (2009). Factor H facilitates the clearance of GBM bound iC3b by controlling C3 activation in fluid phase. Mol Immunol.

[35] Fakhouri F, de Jorge EG, Brune F, Azam P, Cook HT, Pickering MC (2010). Treatment with human complement factor H rapidly reverses renal complement deposition in factor H-deficient mice. Kidney Int.

[36] Sirmaci A, Walsh T, Akay H (2010). MASP1 mutations in patients with facial, umbilical, coccygeal, and auditory findings of Carnevale, Malpuech, OSA, and Michels syndromes. Am J Hum Genet.

[37] Degn SE, Jensenius JC, Thiel S (2013). Response to Comment on ‘Mannan-binding lectin-associated serine protease (MASP)-1 is crucial for lectin pathway activation in human serum, whereas neither MASP-1 nor MASP-3 is required for alternative pathway function’. J Immunol.

[38] Takahashi M, Sekine H, Endo Y, Fujita T (2013). Comment on ‘Mannan-binding lectin-associated serine protease (MASP)-1 is crucial for lectin pathway activation in human serum, whereas neither MASP-1 nor MASP-3 is required for alternative pathway function’. J Immunol.

[39] Rooryck C, Diaz-Font A, Osborn DP (2011). Mutations in lectin complement pathway genes COLEC11 and MASP1 cause 3MC syndrome. Nat Genet.

[40] Takahashi M, Endo Y, Fujita T (2012). Developmental abnormalities in Masp1/3-deficient mice. Immunol.

